# Discarding duplicate ditags in LongSAGE analysis may introduce significant error

**DOI:** 10.1186/1471-2105-8-92

**Published:** 2007-03-14

**Authors:** Jeppe Emmersen, Anna M Heidenblut, Annabeth Laursen Høgh, Stephan A Hahn, Karen G Welinder, Kåre L Nielsen

**Affiliations:** 1 Department of Biotechnology, Chemistry and Environmental Engineering, Aalborg University, Aalborg, Denmark; 2 Department of Internal Medicine, Knappschaftskrankenhaus, University of Bochum, Bochum, Germany; 3 Department of Molecular Oncology, Weatherall Institute of Molecular Medicine, Oxford University John Radcliffe Hospital, UK

## Abstract

**Background:**

During gene expression analysis by Serial Analysis of Gene Expression (SAGE), duplicate ditags are routinely removed from the data analysis, because they are suspected to stem from artifacts during SAGE library construction. As a consequence, naturally occurring duplicate ditags are also removed from the analysis leading to an error of measurement.

**Results:**

An algorithm was developed to analyze the differential occurrence of SAGE tags in different ditag combinations. Analysis of a pancreatic acinar cell LongSAGE library showed no sign of a general amplification bias that justified the removal of all duplicate ditags. Extending the analysis to 10 additional LongSAGE libraries showed no justification for removal of all duplicate ditags either. On the contrary, while the error introduced in original SAGE by removal of naturally occurring duplicate ditags is insignificant, it leads to an error of up to 3 fold in LongSAGE. However, the algorithm developed for the analysis of duplicate ditags was able to identify individual artifact ditags that originated from rare nucleotide variations of tags and vector contamination.

**Conclusion:**

The removal of all duplicate ditags was unfounded for the datasets analyzed and led to large errors. This may also be the case for other LongSAGE datasets already present in databases. Analysis of the ditag population, however, can identify artifact tags that should be removed from analysis or have their tag count adjusted.

## Background

Serial Analysis of Gene expression (SAGE) is a global and digital gene expression profiling method [1,2]. It relies on three fundamental principles: (i) a short nucleotide tag cut from a cDNA copy of an mRNA is sufficient to uniquely identify the transcript, (ii) two tags can be ligated together to form ditags and unambiguously amplified by PCR, and (iii) multiple tags can be concatenated for efficient detection by DNA sequencing. The overall reliability of SAGE has been compared to other gene expression profiling methods such as Northern Blots [3], real-time or kinetic PCR[4,5], and cDNA and oligo nucleotide micro array hybridizations [6-8]. It was generally found that the reliability and reproducibility of SAGE is high. Typically 70–85% of gene expression changes observed in SAGE can be confirmed by a different method [4,7]. However, a potential bias introduced by amplification of ditags was discussed already in the original SAGE publication [1]. It was suspected that duplicate ditags, i.e. identical copies of a ditag (AB), would occur only as an artifact of PCR amplification. Therefore, duplicate ditags have been removed prior to tag counting in most SAGE studies so far, partly because of requests from reviewers before publication.

However, duplicate ditags will be encountered naturally with a certain frequency, depending on abundance of the two transcripts from which the ditag is derived [8,9]. For example, in the original SAGE protocol two blunt ended 14 nucleotide tags were ligated to form ditags. Two tags A and B, each occurring at a frequency of 0.02 have a 0.0004 probability of being joined. Present SAGE studies typically include 50,000 tags (25,000 ditags) leading to 10 AB+BA ditags. However, the total count of a tag of 0.02 frequency in 50,000 is 1000, and the error of 10 introduced by removing the naturally occurring ditags is insignificant. Furthermore, an algorithm to minimize this problem (SAGEparser) was developed by Snyder and coworkers[10].

However, recent developments in SAGE technology have accentuated the problem of discarding duplicate ditags. First, there has been a drive towards using smaller samples for construction of SAGE libraries, facilitating the analysis of cells with specialized functions such as pancreatic cells obtained from biopsies [4]. Such samples may have extreme gene expression profiles with single transcripts accounting for 5 % of the total population of transcripts. Second, the widespread use of the LongSAGE protocol in which a two base pair overhang is used in the ligation of ditags, instead of blunt ends [2]. Consequently, any LongSAGE tag can only form ditags with tags with a compatible overhang, in principle reducing the number of potential partner tags 16 fold on the average. In this paper we analyze the error introduced by discarding naturally occurring duplicate ditags in LongSAGE and describe a probabilistic algorithm that can distinguish naturally occurring ditags from artifacts.

## Results and discussion

**Figure 1:**
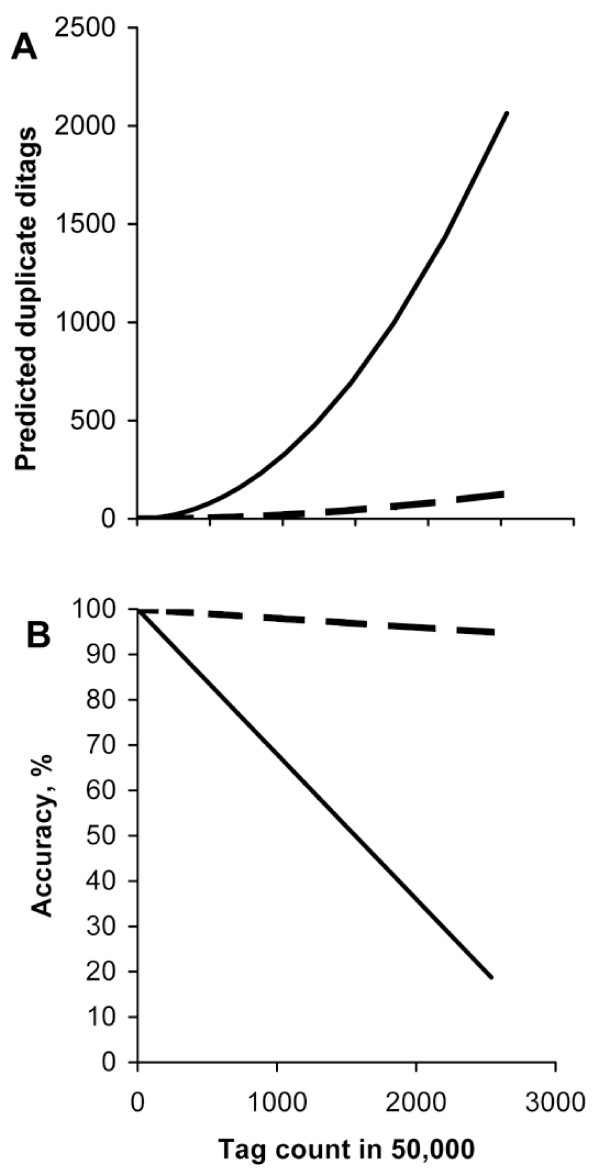
**Estimating occurrence of duplicate ditags in SAGE based on an even distribution of compatible overlapping tags**. **(A) **The number of expected duplicate ditags (equation 1). (B) The accuracy of tag count when duplicate ditags were removed from the analysis. SAGE (hatched line), and LongSAGE (solid line).

A prediction of the number of duplicate ditags as a function of the abundance of the two monotags in SAGE and LongSAGE is shown in figure 1. It illustrates the error introduced by deleting duplicate ditags as commonly practiced in these analyses. In original SAGE, all blunt-ended tags can combine with all other tags and, therefore, the number of a particular duplicate ditag AB can be predicted from equation 1. In LongSAGE, tags have a 2 nt overhang and their ditag partner is constrained accordingly. Therefore, the number of a particular LongSAGE ditag can be estimated from equation 2, assuming an even distribution of possible overhangs.

As can be seen in figure 1, discarding duplicate ditags introduces a serious error for abundant tags in LongSAGE data analysis. For example, two abundant tags, each present 1500 times in a typical 50,000 tag study, would give rise to only 45 duplicate ditags in SAGE, but 720 duplicate ditags in LongSAGE. Counting duplicates only once, as is presently done, would result in an error of 3% for SAGE and 48% for LongSAGE. In this example we have removed the duplicates for AB only, but in reality a particular tag A, will create duplicates with any compatible tag B, C, D etc. at the frequency stipulated in equations 1 or 2. If each of these duplicates is counted only once, a further reduction of the tag count is introduced and, therefore, the error will increase. Indeed, this simple estimate demonstrates that the problem is expected to be much greater for LongSAGE, than for conventional SAGE.

However, the assumption of equal proportions of compatible overhangs in LongSAGE is unrealistic. The genome sequence is not a random distribution of nucleotides [11] and furthermore, this would limit the maximum tag count of any tag to 1/16 of the total tag count of the library (e.g. 3125 in 50,000), and individual tag counts larger than 3125 have been encountered.

The experimental dataset derived from RNA isolated from pancreatic acinar cells by the aRNA-LongSAGE procedure was therefore analyzed in greater detail [4]. It contains 44,276 tags before removal of duplicate ditags and 31,868 after. The unusual high numbers of duplicate ditags reflects an extraordinary abundance of transcripts encoding the enzymes of the digestive juice (table 1). Table 1 shows the 12 most abundant tags, the enzyme encoded and the tag counts before and after removal of duplicate ditags. Removing duplicate ditags from the dataset reduces the tag count with up to 67% for the most abundant tag. The most abundant tags are the ones most often affected, although medium abundance tags are also significantly affected, if they are compatible with predominant tags (see table 2, and additional file 1). In fact, only for total tag counts (without removal of duplicate ditags) less than 10 is the majority of tags unchanged by removal of duplicate ditags. And 19% of medium abundance tags (20–49) are changed at least 1.5 fold. Bear in mind, that the error is not affecting all tags to a similar degree, while some tags are unchanged, others may be greatly affected. For an example, the tag count for CATGGGCGACTCTGGCGGCCC is 40 tags before removal and only 14 after, a change of 3 fold [additional file 1]. Anisimov *et al*. have argued that up to 5% false ditags, so-called quasi-ditags should be removed from SAGE analyses [12]. In our case, removing quasi-ditags has only marginal effect of the analysis (data not shown), presumably because the error corrected by Anisimov *et al*. is exclusively affecting rare tags, whereas we are concerning with an error increasingly affecting tags the more abundant they get.

**Table 1:** Abundant LongSAGE tags observed in pancreatic acinar cells.

	Duplicate ditags				
	Included^a^	Removed^b^			
Tag sequence	Tag count	Tag count	Fold change^c^	THC^d^	Gene name
CATGTCAGGGTGATTCTGGTG	3315	1086	0.33	2531342	Trypsin I
CATGGCGTGACCAGCTTTGTT	2609	1161	0.44	2498325	Elastase IIIB
CATGAATTGAAGAAACTGACC	2359	713	0.30	2510696	Unknown
CATGGAGCACACCCTGAATCA	1145	657	0.57	2613307	Carboxypeptidase A1
CATGGAACACAAAAAAAAAAA	1094	535	0.49		Unknown
CATGTGCGAGACCACCCCTAT	891	461	0.52	2683646	Carboxypeptidase A2
CATGTCCTCAAAACAAAAAAA	753	377	0.50		Unknown
CATGAGCCTTGGTATCAAGAG	645	353	0.55	2462969	Cholesterol esterase
CATGTTCATACACCTATCCCC	531	177	0.33	2398611	NADH dehydrogenase
CATGCTGAATCTAAATTATAA	526	257	0.49	2590573	Alpha-amylase 2B
CATGTCCTCAAAACAATAAAA	465	252	0.54		Unknown
CATGTCCTCAAAAAAAAAAAA	431	211	0.49		Unknown

^a^Total number of tags = 44,276

^b^Total number of tags = 31,868

^c^tag count including duplicate ditags/tag count excluding duplicate ditags.

^d ^Tentative Human Contig number from The Institute for Genomic Research

**Table 2:** The relationship of tag abundance and degree of change introduced by removal of duplicate ditags.

Tag count	# of unique tags	Observed change upon removal of duplicate ditags			
		
		>2 fold^a^	1.5–2 fold^a^	1–1.5 fold^a^	unchanged^a^
>200	19	7 (37)	8 (42)	4 (21)	0 (0)
>100–199	13	3 (23)	4 (31)	6 (46)	0 (0)
>50–99	42	2 (5)	13 (31)	27 (64)	0 (0)
>20–49	91	6 (7)	11 (12)	72 (79)	2 (2)
>10–19	179	3 (2)	16 (9)	104 (58)	56 (31)
>5–9	383	1 (0.3)	19 (5)	139 (36)	224 (58)
>2–5	2157	8 (0.4)	240 (11)	120 (6)	1789 (83)

^a ^Fold change is calculated by dividing the total tag count with the tag count obtained after removal of duplicate ditags. Percentage of total number of different tags in the indicated intervals is given in parentheses.

The corrective measures suggested by Welle [9]and Snyder [10] are both iterative approaches developed for SAGE. Welle suggests splitting up the dataset in a number of sub-datasets, removing duplicate ditags, and then adding these datasets together allowing duplicates. The number of subdatasets to be created, determining the maximal number of duplicate ditags is a simple guess. In fact, this method is equivalent to setting a maximal allowed ditag count instead of excluding all. In LongSAGE, duplicate ditag counts of several hundreds are frequently observed. Therefore, determining a low meaningful fixed number of duplicate ditags is not feasible. Snyder's algoritm (SAGEparser) includes a proportion of the observed duplicate ditags based on the abundance of the two monotags comprising the ditag. This new tag count can then be used to calculate a new proportion of the observed duplicate ditags to be added. Many iterations of this algorithm would approach the inclusion of all duplicate ditags. While this algorithm includes some of the naturally occurring ditags, it only works for SAGE where all tags can form ditags with each other and does not address whether an entire library is biased or not.

In this study, an algorithm implemented in Perl was developed (LongSAGE_bias.pl, see methods for details) which extracts both monotags and ditags from phred or fasta formatted sequence files, defines the two nt overhang of tag pairs in the ditags, and counts and sorts these ditags into compatible overlapping classes. Of the 44,276 tags in total, 34,464 were seen twice or more. Considering these tags only (thus excluding most tags originating from sequencing error) 12,408 (36%) were present in duplicate ditags. A major complication of the analysis is the presence of most abundant tags in several forms differing in length by one or rarely by two nucleotides. Thus a single tag may be split into two or more compatible overlapping classes. For this analysis, only tags between 40 and 42 nt were considered (including the NlaIII recognition site, CATG of both tags). These accounted for 98.6% of all ditags (table 3). A 40 nucleotide ditag contains two tags in the short form (21 nt each, as 2 nt are shared), a 41 nucleotide ditag one short form and one long form, and a 42 nucleotide ditag contains two tags in the long form (22 nt each, as 2 nt are shared). The relative propensity for a tag to appear in long or short form was calculated for each tag without considering the 41 nucleotide ditag, as for these ditags, we cannot know which of the two tags is present in the long form. Likewise, the compatible overlapping class was determined from the 40 and 42 nucleotide ditags only, because the overlap in these tags can be unambiguously identified as the two central nucleotides of the ditag.

**Table 3:** Summary of ditag statistics.

	Pancreatic acinar cells	RefSeq v.16^c^
Ditag length^a^	Number	

40	3339	N.A.
41	11329	N.A.
42	7325	N.A.
43	240	N.A.
44	61	N.A.

Overlap class	Number	Number

AT^b^	572	5172
CG^b^	17	1371
GC^b^	344	3635
TA^b^	161	5076
AA or TT	3173	18853
AC or GT	1784	8120
AG or CT	813	11069
CA or TG	437	10467
CC or GG	2868	8561
GA or TC	495	9315

^a^Ditags shorter than 40 nucleotides were not extracted from sequences

^b^Palindromic sequences. The number of sequences compares to half the number in non-palindromic sequences.

^c^LongSAGEtags generated in-silico form RefSeq using 17+CATG nt tags only.

The number of ditags in the 10 possible overlapping classes is tabulated in table 3. The length distribution shows a general overrepresentation of the long form. Apparently, the tag generating restriction enzyme, MmeI, cleaves the DNA strand 21/19 nucleotides downstream of its recognition site twice as often as 20/18 nucleotides. Furthermore, the true distribution among compatible overlapping classes is far from uniform. The overlap CG was only present in 17 ditags, whereas the AA (or TT) was present in 3173 ditags. This is in line with the relative dinucleotide abundance in humans, which also shows a severe under representation of CG, whereas AA (or TT) is more commonly observed [11]. This observation is corroborated with the finding that CpG islands are predominantly found in the first exon of genes and therefore rarely in the 3' end of transcripts mostly represented in SAGE [13]. Analyzing the distribution of overlaps generated *in-silico *from the human RefSeq v. 16 also shows a similar tendency, albeit to a lesser extent (table 3).

The uneven distribution of compatible overhangs actually raises the question whether a particular overhang can be present in such a low abundance that it suppresses the measurement of other tags due to an insufficient number of compatible monotags. One solution to this would be to conduct the LongSAGE experiments by blunt-ending by T4-DNA polymerase prior to ligation of ditags, thus shortening the LongSAGE tags by two nucleotides [14]. An advantage of this approach is that the error introduced by removal of duplicate ditags is small and similar to those indicated for SAGE (figure 1). However, in the case presented here, saturation of compatible overlaps is not the reason for the rare occurrence of the CG overlap, as CG is a palindrome and thus may form ditags with itself.

**Figure 2:**
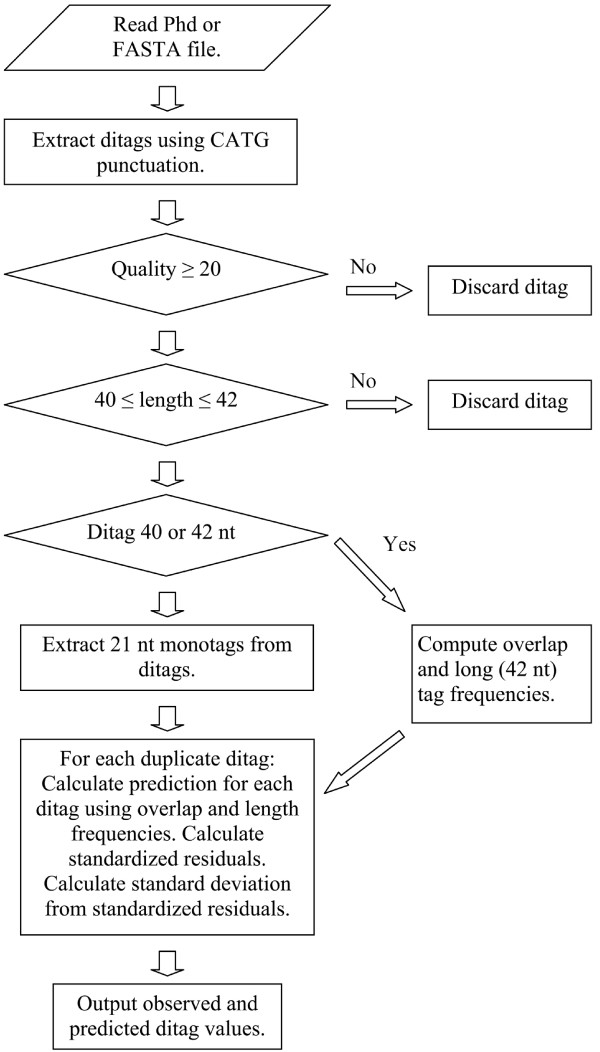
**Overview of the LongSAGE_bias.pl PERL script used for the data analyses**. The quality threshold of sequence files can be set at any level desired. A high quality threshold may lead to the under representation of difficult to sequence tags. If set to zero all tags are included and the number of tags observed once or twice increases.

**Figure 3:**
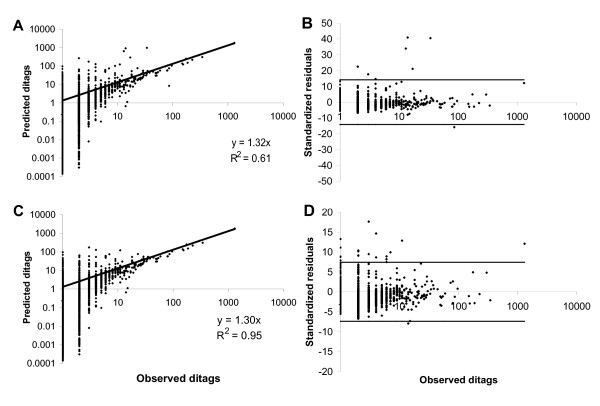
**Predicted versus observed duplicate ditags in a LongSAGE study of pancreatic acinar cells [4]**. Predicted ditag counts, two for each observed ditag, were calculated according to equation 3 (see methods for details). (A) All observed ditags are included. (C) Outliers, according to table 4 were removed. Standardized residuals were calculated according to equation 4. The confidence interval at three standard deviations is shown as lines. (B) All observed ditags are included. (D) Outliers, according to table 4 were removed. The recalculated confidence interval at three standard deviations is shown as lines.

Two predictions are calculated for the occurrence of each ditag (including 41 nucleotide ditags) using equation 3 (see methods and figure 2 for details). The two predictions are often the same, but may differ for ditags containing less abundant tags and will approach uniformity for higher duplicate ditag counts. A plot of the predicted numbers of ditags against the observed number of ditags is shown in figure 3a [see additional file 2 for details]. The slope of a linear regression analysis of the data is 1.3, in reasonable agreement with the expected 1. However, the Pearson product moment correlation coefficient between the observed and the predicted is a modest 0.61.

Detection of outliers is performed by calculating standardized residuals according to equation (4) for each ditag. Assuming normal distribution of the standardized residuals, the standard deviation is calculated for all ditags observed more than once. An observation is classified as an outlier if the standardized residual is larger than three standard deviations (99% confidence). The standardized residuals are plotted in figure 3b. The low number of standardized residuals that fall outside the confidence interval (outliers) indicates that most duplicate ditags seem to represent true tags.

Inspection of the outliers reveals that most of these ditags contain at least one tag ending in a nucleotide that is inconsistent with the nucleotide sequences of the corresponding Unigene found in the TIGR Human Gene Index (see table 4), and most likely represents nucleotide variations. The overlapping class of this variant tag is inconsistent with the overlapping class of the non-variant tag used for the prediction. Obviously, the algorithm would provide erroneous predictions in these cases, since it is based on the frequency of the non-variant monotag, which is much higher than the frequency of the variant monotag. A different, rather abundant ditag was observed (86 times) much more often than predicted (8 times). BLAST analysis of this ditag reveals that it consists of two tags derived from the *E. coli *β-lactamase gene and thus is most likely the result of vector contamination. Removing these data points from the regression increases the Pearson correlation coefficient to 0.95. Therefore, an excellent correlation between the number of duplicate ditags observed and predicted is obtained not discarding duplicate ditags.

**Table 4:** Comparison of outlier ditags with the matched database sequences.

**Obs**	**Pred**	**Tag structure**	**THC**^a^	**Gene name**
34	970	CATGGAGCACACCCTGAATCACACCAGAATCACCCTGACATG		
		CATGGAGCACACCCTGAATCAC	2401106	Carboxypeptidase
		**C**CACCAGAATCACCCTGACATG	2434341	Trypsin I
17	289	CATGGTGTGTGCTGGAGGGTACACCAGAATCACCCTGACATG		
		CATGGTGTGTGCTGGAGGGTAC	2431718	Elastase IIIA
		**C**CACCAGAATCACCCTGACATG	2434341	Trypsin I
14	913	CATGTCAGGGTGATTCTGGTGAGGAAGCCCACACAGAACATG		
		CATGTCAGGGTGATTCTGGTG**G**	2434341	Trypsin I
		**A**AGGAAGCCCACACAGAACATG	2434342	Trypsin I
13	641	CATGACGCTGGACGCTCCAAGCACCAGAATCACCCTGACATG		
		CATGACGCTGGACGCTCCAAGC	2407612	Colipase
		**C**CACCAGAATCACCCTGACATG	2434341	Trypsin I
9	101	CATGTCAGGGTGATTCTGGTGTGATTGCCGAGCCAGAGCATG		
		CATGTCAGGGTGATTCTGGTG**G**	2434341	Trypsin I
		GTGATTGCCGAGCCAGAGCATG	2237360	Phospholipase A2^b^
4	127	CATGTCAGGGTGATTCTGGTGCTGGCGCTTCTGACCATCATG		
		CATGTCAGGGTGATTCTGGTG**G**	2434341	Trypsin I
		GCTGGCGCTTCTGACCATCATG	2401106	Carboxypeptidase ^c^
4	85	CATGACGCTGGACGCTCCAAGTGATTCAGGGTGTGCTCCATG		
		CATGACGCTGGACGCTCCAAG**C**	2407612	Colipase
		GTGATTCAGGGTGTGCTCCATG	2401106	Carboxypeptidase
2	267	CATGGAGCACACCCTGAATCAAACAAAGCTGGTCACGCCATG		
		CATGGAGCACACCCTGAATCA**C**	2401106	Carboxypeptidase
		AAACAAAGCTGGTCACGCCATG	2254617	Elastase IIIB
86	8	CATGACAGTAAGAGAATTATGCAGTGCTGCCATAACCATG		
		CATGACAGTAAGAGAATTATGC		β-lactamase
		GCAGTGCTGCCATAACCATG		Inv. β-lactamase

^a^Tentative Human Contig number from The Institute for Genomic Research (TIGR).

^b^The match to Phospholipase A2 is not perfect (GTGATTGCCGAGCCAGAGCA**C**G)

^c^The tag matches an inverted sequence from carboxypeptidase.

To investigate whether this is special to this particular dataset, we have performed the analysis on additional datasets, five derived from potato tuber (Høgh, Emmersen and Nielsen, unpublished) and five other libraries derived from pancreatic tissue [4]. The analysis can be carried out on any LongSAGE library, but becomes more precise with more ditags included in the analysis. In our experience, depending on the proportion of duplicate ditags present, a minimum library size of 35,000 tags seems to be the lower limit for a reliable analysis. In the future, exploiting new DNA sequencing technologies, libraries larger than 150,000 tags will probably be common[15]. Varying numbers of duplicate ditags were present in these libraries, and suspicious outlier ditags were identified in all libraries. However, in none of the libraries the duplicate ditags were biased to an extent that justified the bulk removal of all duplicates. Tag extractions including or excluding duplicate ditags of the most abundant transcripts of all additional 10 libraries analyzed is shown in [additional file 3]. The libraries are affected to a different extent depending on the transcription profile, but all libraries show a change of up to 1.5–2 folds among the 20 most abundant transcripts, when including or excluding duplicate ditags [see additional file 3]. However, similar to the pancreatic acinar library, the effect was not restricted to high abundance tags, but was observed in medium abundance tags as well (data not shown). The NCBI database, SAGEdB, currently contains 625 SAGE libraries of which 155 are LongSAGE. Unfortunately, ditag sequences are not reported so re-analysis of existing data will have to be carried out by the submitters which hold the original sequence files.

Also, it is important to consider how the removal of duplicate ditags influences the initial identification of a gene as regulated in a comparison of two transcript profiles. To assess whether this changes by exclusion of duplicate ditags, we compared the pancreatic acinar library with one derived from pancreatic ductal cells with and without the inclusion of duplicate ditags. Excluding duplicate ditags, 122 tags was identified as statistically significantly regulated (P < 0.05 with Bonferroni correction). Including duplicates yielded 56 new tags, while three fell below the statistical cut-off (additional 43%) (See table 5). Some of the tags mapped to genes that are known to be highly expressed in acinar but not ductal cells, such as variants of the digestive enzymes chymotrypsin, trypsin and elastase. Therefore, removal of duplicate ditags alters the interpretation of LongSAGE data at least by limiting the power of detecting changes in gene expression; thus effectively excluding what is likely to be valid transcript changes (false negatives) from further analysis and interpretation.

**Table 5:** Additional transcript changes detected between pancreatic acinar and ductal cells by including duplicate ditags.

**Tag**	**Acinar**	**Ductal**	**P-value**	**Transcript ID**^a^	**Gene Name**
CATGGGCGACTCTGGCGGCCC	40	0	6.72E-13	THC2268952	Chymotrypsinogen B
CATGGAGCACACCCTGAATCC	39	0	1.4E-12	unknown	
CATGCCTGTAATCCCAGCTAC	20	95	1.23E-11	W85818	
CATGCCTAGCTGGATTGCAGA	26	104	5.89E-11	BU542624	
CATGAAAGTCTAGAAATAAAA	3	41	3.62E-09	THC2400275	full-length cDNA clone CS0DC017YH08
CATGCACAAACGGTAGTTTTG	187	110	3.78E-09	AV744668	
CATGTGTGCTAAATGTGTTCG	69	22	5.56E-09	BF089871	
CATGTTCTGTGTGGGCTTCCC	27	0	8.83E-09	unknown	
CATGTGCATCTGGTGTAGGAA	33	103	1.49E-08	BU626127	
CATGGGGTTGGCTTGAAACCA	2	35	1.72E-08	BG756271	
CATGCACCTCCCACCGGCCGT	26	0	1.82E-08	THC2457279	Elastase 2B
CATGCTAAGACTTCACCAGTC	58	145	2.1E-08	BU674671	
CATGGTAAGTGTACTGGAAAG	33	4	3.3E-08	THC2400569	Human mitochondrial genes
CATGAATCCTTGCCTCCCTCA	25	1	3.74E-08	BI791939	
CATGGGAACAAACAGATCGAA	6	44	6.7E-08	NP922813	CD24 protein
CATGGTAATTTAAACAATGAA	0	29	7.31E-08	THC2336784	Integrin beta-6 precursor
CATGTCCCCGTGGCTGTGGGG	1	29	7.31E-08	AV700058	
CATGTGCCCTCAGGAAAAAAA	0	29	7.31E-08	THC2244374	Neutrophil gelatinase-associated lipocalin
CATGGAACACAAAAAAAAAGA	24	0	7.68E-08	unknown	
CATGTGGCTTCAAGCCACCAG	28	89	8.5E-08	BF987687	
CATGCCAAACGTGTAACAATT	7	46	8.61E-08	CV350470	
CATGACAGTAAGAGAATTATG	87	39	1.11E-07	unknown	
CATGCTGTACAGACACCACCA	0	28	1.33E-07	BG151226	
CATGGTAAATTTAAAAAAAAA	1	28	1.33E-07	unknown	
CATGAGTTGAAGAAACTGACC	23	0	1.57E-07	unknown	
CATGGTTATGGCAGCACTGCA	86	39	1.64E-07	unknown	
CATGGGTGGTGTCTGAGAGGC	0	27	2.41E-07	THC2256155	gastrointestinal glutathione peroxidase 2
CATGTTCATTATAATCTCAAA	8	46	2.72E-07	BG025220	
CATGCATCTTCACCAGCAGCT	4	36	2.75E-07	CD240368	
CATGCTGCTTGGTGAACAATC	4	36	2.75E-07	THC2247807	Neutral and basic amino acid transport protein
CATGTATGACTTAATAAATCC	2	30	3.01E-07	AA506911	
CATGCTTGTGAACTGCACAAC	0	26	4.38E-07	AA343639	
CATGGAAATTTAAAGCAGGTT	2	29	5.31E-07	THC2272041	
CATGCCAGAACAGACTGGTGA	19	67	5.89E-07	CD240292	
CATGCCAGGGTGATTCTGGTG	21	0	6.58E-07	THC2434375	Trypsin II
CATGGTGTGCGCTGGGGGCGT	21	0	6.58E-07	unknown	
CATGGATTGAAGAAACTGACC	21	0	6.58E-07	unknown	
CATGTGTCCACCATCTCTCTG	21	0	6.58E-07	THC2434352	Trypsinogen C
CATGGCGTGACCAGCTTTGTG	21	1	6.58E-07	unknown	
CATGAGCCACTGCGCCCAGCC	26	3	6.96E-07	H75720	
CATGCTTCTGATCTCAGCAGT	0	25	7.92E-07	THC2315603	Heparan sulfate 3-O-sulfotransferase-1
CATGCACAGGCAAAATGTATT	1	25	7.92E-07	CA314838	
CATGTGAAGTTATACTGTGGC	2	28	9.33E-07	AW970111	
CATGGGATATGTGGTGTATAT	7	41	9.67E-07	AV656761	
CATGCATATCATTAAACAAAT	5	36	0.00000106	NP924865	Insulin-like growth factor binding protein 7
CATGTATTTTCCAGCTGCCTC	20	1	0.00000134	AA514440	
CATGTCAGGGTGGTTCTGGTG	20	1	0.00000134	unknown	
CATGTCAGGGTGATCCTGGTG	20	0	0.00000134	unknown	
CATGTCAGGGCGATTCTGGTG	20	0	0.00000134	unknown	
CATGAAAAGCAGAAATCGGTT	0	24	0.00000143	THC2244965	Krueppel-like factor 5
CATGTTTGCACCTTTCTAGTT	0	24	0.00000143	NP119453	Connective tissue growth factor
CATGATACTTTAATCAGAAGC	1	24	0.00000143	NP1194136	full-length cDNA clone CS0DF028YA19
CATGGCGAAACCCTGTCTCTA	3	30	0.00000155	W03579	
CATGCTTATGGTTGATCAGTT	2	27	0.00000164	CB243786	
CATGCACCTAATTGGAAGCGC	56	22	0.00000216	CF129138	
CATGGGAATGTACGTTATTTC	13	52	0.00000218	NP1215187	mitochondrial ATP synthase

^a^The percentage of tags idenfied as differentially expressed that cannot be matched to database sequences are similar including (27%) or excluding (30%) ditags as well as in this list (24%).

It has been found, that the cross-platform agreement of transcriptome measurements by SAGE, LongSAGE and DNA oligonucleotide microarrays was only modest, in contrast to a good intra-platform reproducibility [7]. A majority, but not all of the differentially expressed genes identified by either method can be verified by RT-PCR (e.g. [4] and [16]). Therefore, to maximize the accuracy of LongSAGE and hopefully improve the cross-platform consistency, it is important analyze and adjust the tag sequences (including all duplicate ditags) for a number of potential biases prior to mapping to database sequences. First, linker sequences should be removed. Second, the dataset should be tested with the present algorithm. Duplicate ditag counts that falls within the confidence intervals should not be adjusted. But in the case of outliers, these can be manually removed after careful analysis (as in this study), or automatically adjusted to the predicted count by the algorithm to minimize the impact of artifacts. Third, to minimize the number of false positives identified as differentially expressed genes, artifact tags originating from sequence errors should be resolved using SAGEScreen [17].

## Conclusion

The analyses presented here clearly demonstrate that the present procedure of discarding all duplicate ditags can lead to large errors in LongSAGE studies. Instead, the algorithm described here should be used to test LongSAGE datasets and identify potentially biased ditags that should be adjusted or removed. Based on the results obtained it is likely, that most of the transcriptome profiles present in the databases have been artificially biased by the removal of duplicate ditags.

## Methods

### Equations

Assuming that the observed tag counts (after amplification and sequence extraction) are representative of the actual distribution of tag molecules, the expected occurrence of a duplicate ditag AB in SAGE can be approximated by

DAB, pred=Dtotal∗P(AB)= Dtotal∗P(A)∗P(B)= Dtotal∗TATtotal∗TBTtotal     (1)
 MathType@MTEF@5@5@+=feaafiart1ev1aaatCvAUfKttLearuWrP9MDH5MBPbIqV92AaeXatLxBI9gBaebbnrfifHhDYfgasaacH8akY=wiFfYdH8Gipec8Eeeu0xXdbba9frFj0=OqFfea0dXdd9vqai=hGuQ8kuc9pgc9s8qqaq=dirpe0xb9q8qiLsFr0=vr0=vr0dc8meaabaqaciaacaGaaeqabaqabeGadaaakeaacqqGebardaWgaaWcbaGaeeyqaeKaeeOqaiKaeeilaWIaeeiiaaIaeeiCaaNaeeOCaiNaeeyzauMaeeizaqgabeaakiabg2da9iabbseaenaaBaaaleaacqqG0baDcqqGVbWBcqqG0baDcqqGHbqycqqGSbaBaeqaaOGaey4fIOIaeeiuaa1aaSbaaSqaaiabbIcaOiabbgeabjabbkeacjabbMcaPaqabaGccqGH9aqpcqqGGaaicqqGebardaWgaaWcbaGaeeiDaqNaee4Ba8MaeeiDaqNaeeyyaeMaeeiBaWgabeaakiabgEHiQiabbcfaqnaaBaaaleaacqqGOaakcqqGbbqqcqqGPaqkaeqaaOGaey4fIOIaeeiuaa1aaSbaaSqaaiabbIcaOiabbkeacjabbMcaPaqabaGccqGH9aqpcqqGGaaicqqGebardaWgaaWcbaGaeeiDaqNaee4Ba8MaeeiDaqNaeeyyaeMaeeiBaWgabeaakiabgEHiQmaalaaabaGaemivaq1aaSbaaSqaaiabdgeabbqabaaakeaacqWGubavdaWgaaWcbaGaemiDaqNaem4Ba8MaemiDaqNaemyyaeMaemiBaWgabeaaaaGccqGHxiIkdaWcaaqaaiabdsfaunaaBaaaleaacqWGcbGqaeqaaaGcbaGaemivaq1aaSbaaSqaaiabdsha0jabd+gaVjabdsha0jabdggaHjabdYgaSbqabaaaaOGaaCzcaiaaxMaadaqadaqaaiabigdaXaGaayjkaiaawMcaaaaa@7FC2@

where D is the number of ditags, P the probability, and T the number of monotags observed.

The expected occurrence of a duplicate ditag AB in LongSAGE, assuming even distribution of compatible overlapping classes is then (including duplicate ditags).

DAB,pred=Dtotal∗TATtotal∗TBTtotal∗16     (2)
 MathType@MTEF@5@5@+=feaafiart1ev1aaatCvAUfKttLearuWrP9MDH5MBPbIqV92AaeXatLxBI9gBaebbnrfifHhDYfgasaacH8akY=wiFfYdH8Gipec8Eeeu0xXdbba9frFj0=OqFfea0dXdd9vqai=hGuQ8kuc9pgc9s8qqaq=dirpe0xb9q8qiLsFr0=vr0=vr0dc8meaabaqaciaacaGaaeqabaqabeGadaaakeaacqqGebardaWgaaWcbaGaeeyqaeKaeeOqaiKaeeilaWIaeeiCaaNaeeOCaiNaeeyzauMaeeizaqgabeaakiabg2da9iabbseaenaaBaaaleaacqqG0baDcqqGVbWBcqqG0baDcqqGHbqycqqGSbaBaeqaaOGaey4fIOYaaSaaaeaacqqGubavdaWgaaWcbaGaeeyqaeeabeaaaOqaaiabbsfaunaaBaaaleaacqqG0baDcqqGVbWBcqqG0baDcqqGHbqycqqGSbaBaeqaaaaakiabgEHiQmaalaaabaGaeeivaq1aaSbaaSqaaiabbkeacbqabaaakeaacqqGubavdaWgaaWcbaGaeeiDaqNaee4Ba8MaeeiDaqNaeeyyaeMaeeiBaWgabeaaaaGccqGHxiIkcqqGXaqmcqqG2aGncaWLjaGaaCzcamaabmaabaGaeeOmaidacaGLOaGaayzkaaaaaa@5D8B@

The expected occurrence of a duplicate ditag AB in LongSAGE, using dataset specific distributions of compatible overlapping classes can be approximated by

DAB=Dtotal∗TA/Ttotal∗TBTPPT     (3)
 MathType@MTEF@5@5@+=feaafiart1ev1aaatCvAUfKttLearuWrP9MDH5MBPbIqV92AaeXatLxBI9gBaebbnrfifHhDYfgasaacH8akY=wiFfYdH8Gipec8Eeeu0xXdbba9frFj0=OqFfea0dXdd9vqai=hGuQ8kuc9pgc9s8qqaq=dirpe0xb9q8qiLsFr0=vr0=vr0dc8meaabaqaciaacaGaaeqabaqabeGadaaakeaacqqGebardaWgaaWcbaGaeeyqaeKaeeOqaieabeaakiabg2da9iabbseaenaaBaaaleaacqqG0baDcqqGVbWBcqqG0baDcqqGHbqycqqGSbaBaeqaaOGaey4fIOYaaSaaaeaacqqGubavcqqGbbqqcqqGVaWlaeaacqqGubavdaWgaaWcbaGaeeiDaqNaee4Ba8MaeeiDaqNaeeyyaeMaeeiBaWgabeaaaaGccqGHxiIkdaWcaaqaaiabbsfaujabbkeacbqaaiabbsfaunaaBaaaleaacqqGqbaucqqGqbaucqqGubavaeqaaaaacaWLjaGaaCzcamaabmaabaGaeG4mamdacaGLOaGaayzkaaaaaa@5179@

where T_PPT _is the sum of all possible partner tags.

Standardized residuals was calculated as follows [18]

Y=(Dpred+12)DpredT(Y) =1−Y2+2∗Y∗ln(Y)1−Y2, T(1) = 0StdRes = (Dobs− Dpred+23)1+T(Y)Dpred     (4)
 MathType@MTEF@5@5@+=feaafiart1ev1aaatCvAUfKttLearuWrP9MDH5MBPbIqV92AaeXatLxBI9gBaebbnrfifHhDYfgasaacH8akY=wiFfYdH8Gipec8Eeeu0xXdbba9frFj0=OqFfea0dXdd9vqai=hGuQ8kuc9pgc9s8qqaq=dirpe0xb9q8qiLsFr0=vr0=vr0dc8meaabaqaciaacaGaaeqabaqabeGadaaakeaafaqaaeWabaaabaGaeeywaKLaeyypa0ZaaSaaaeaacqqGOaakcqqGebardaWgaaWcbaGaeeiCaaNaeeOCaiNaeeyzauMaeeizaqgabeaakiabgUcaRmaalaaabaGaeeymaedabaGaeeOmaidaaiabbMcaPaqaaiabdseaenaaBaaaleaacqWGWbaCcqWGYbGCcqWGLbqzcqWGKbazaeqaaaaaaOqaaiabbsfaujabbIcaOiabbMfazjabbMcaPiabbccaGiabg2da9maalaaabaGaeeymaedccaGae8NeI0IaeeywaK1aaWbaaSqabeaacqaIYaGmaaGccqGHRaWkcqqGYaGmcqGHxiIkcqqGzbqwcqGHxiIkcqqGSbaBcqqGUbGBcqqGOaakcqqGzbqwcqqGPaqkaeaacqqGXaqmcqWFsislcqqGzbqwdaahaaWcbeqaaiabikdaYaaaaaGccqqGSaalcqqGGaaicqqGubavcqqGOaakcqqGXaqmcqqGPaqkcqqGGaaicqWF9aqpcqqGGaaicqqGWaamaeaacqqGtbWucqqG0baDcqqGKbazcqqGsbGucqqGLbqzcqqGZbWCcqqGGaaicqGH9aqpcqqGGaaidaWcaaqaaiabbIcaOiabbseaenaaBaaaleaacqqGVbWBcqqGIbGycqqGZbWCaeqaaiab=jHiTOGaeeiiaaIaeeiraq0aaSbaaSqaaiabbchaWjabbkhaYjabbwgaLjabbsgaKbqabaGccqGHRaWkdaWcaaqaaiabbkdaYaqaaiabbodaZaaacqqGPaqkaeaadaGcaaqaamaalaaabaGaeGymaeJaey4kaSIaemivaqLaeiikaGIaemywaKLaeiykaKcabaGaemiraq0aaSbaaSqaaiabdchaWjabdkhaYjabdwgaLjabdsgaKbqabaaaaaqabaaaaaaakiaaxMaacaWLjaWaaeWaaeaacqaI0aanaiaawIcacaGLPaaaaaa@9015@

### Ditag analysis

A SAGE experiment is performed by digesting cDNA with the frequent cutting restriction enzyme NlaIII, isolating the most 3' fragment and ligating a linker containing the sequence TCCGAC, which is recognized the restriction enzyme MmeI. Tags are generated by MmeI which cleaves the DNA strand 20/18 nt or 21/19 nt downstream of this sequence. Ligated ditags have the general structure CATGXXXXXXXXXXXXXXXX(X)(Y)YYYYYYYYYYYYYYYYCATG, where X denotes tag A and Y denote the reverse complement of tag B. The parentheses indicate that most tags exist in both a short and a long form. Hence, the ditag AB can have the length 40, 41 or 42 nucleotides. Two central base pairs are common to both tag A and tag B and originate from the overlap used during ligation. The Perl script, LongSAGEbias.pl [additional file 4] was developed and used for the data analysis (freely available at [19]). A schematic representation of the script is shown in figure 2. First, the script extracts both ditags and monotags (21 nt), including possible linker derived tags, from DNA sequence files and the corresponding quality values (*.phd) generated by the Phred base caller [20]. Second, the script then calculates the length of each ditag and uses the 40 nt and 42 nt ditags for the calculation of the distribution of compatible overlapping tags and the propensities that each tag is 21 (short form) or 22 (long form) nucleotides. This information is then used to predict the occurrence of any ditag composed of tags observed in a duplicate ditag by equation 3 according to the tag length consistent with the ditag in question. For 40 or 42 nucleotide ditags, a prediction is made for each of the two monotags constituting the ditag.

In the case of 41 nucleotide ditags, the ditag AB is first analyzed. Since A can exist in a 41 nt ditag both in the long and a short form, two predictions are made and the one closest to the observed is chosen. Then, the ditag BA is considered in an identical manner. The standardized residuals are calculated and the results are written to tabulator separated files easily imported into any spreadsheet for further analysis. Assuming the ditag counts are Poisson distributed, the mean can be estimated as the observed count and the standard deviation as the square root of the observed. The confidence interval of ditag counts can thus be estimated as mean ± 2*standard deviation. For small ditag counts this confidence interval extends below zero. Consequently, the standard deviation of the standardized residuals is calculated from ditags observed four or more times only (4-2*√4 = 0).

The algorithm can be set to include all duplicate ditags, remove all duplicate ditags and adjust the observed ditag counts that fall outside the confidence interval to the prediction value.

In sum, libraries derived from pancreatic acinar cells, ductal cells, and four libraries from different grades of pancreatic intraepithelial neoplasia were analyzed from pancreas. In addition, 5 potato tuber libraries derived from 6 week old minitubers, at harvest, two libraries from 60 days post harvest dormant tubers, and from tuber tissue excised from under an emerging sprout.

### Analyzing dinucleotide overlap distribution of tags generated in-silico

LongSAGE tags of 17 nt + CATG were extracted from the human RefSeq v. 16 fasta file and the dinucleotide overlap distributions determined using the PERL script dinuccount.pl [19].

### Comparison of LongSAGE libraries with and without inclusion of duplicate ditags

LongSAGE tags from libraries generated from all potato and pancreatic tissue were extracted using the Perl script sage-phred.pl. For pancreatic acinar and ductal cells the tags were mapped to the Human Gene Index [21] using sagemap.pl. The two libraries were compared using the Perl script acprob.pl and statistically significant changes using strict Bonferroni correction was recorded including or excluding duplicate ditags. All scripts are available at [19].

## Authors' contributions

JE, AMH and KLN have designed the analysis of duplicate ditags. JE have produced scripts and performed the analysis. SAH and AMH have performed the LongSAGE studies on pancreatic tissue. ALH carried out the LongSAGE analyses on the potato. KLN drafted the manuscript, which was extensively discussed and modified by KLN, AMH, JE and KGW. Finally, all authors read and approved the final manuscript.

## Supplementary Material

Additional File 1 Pancreatic acinar LongSAGE. Additional file 1 contains the tag counts of the pancreatic acinar LongSAGE library described in the manuscript with and without duplicate ditags. Click here for file

Additional File 2 Observed and predicted LongSAGE ditags of pancreatic acinar cells. Additional file 2 contains the data that constitutes figure 3 and is the background for table 1 and 2. Click here for file

Additional File 3 Most abundant transcripts with and without the removal of duplicate ditags. Additional file 3 contains the top20 transcripts of the ten LongSAGE libraries discussed in the text with and without duplicate ditags. Click here for file

Additional File 4 longsagebias.pl. This file contains the PERL script that performs the ditag analysis described. Click here for file
